# Prognostic Value of Tumor-Associated Macrophages According to Histologic Locations and Hormone Receptor Status in Breast Cancer

**DOI:** 10.1371/journal.pone.0125728

**Published:** 2015-04-17

**Authors:** Jae Moon Gwak, Min Hye Jang, Dong Il Kim, An Na Seo, So Yeon Park

**Affiliations:** 1 Department of Pathology, Seoul National University College of Medicine, Seoul, Republic of Korea; 2 Department of Pathology, Seoul National University Bundang Hospital, Seongnam, Gyeonggi, Republic of Korea; 3 Green Cross Laboratory, Yongin, Gyeonggi, Republic of Korea; 4 Department of Pathology, Kyungpook National University Hospital, Kyungpook National University School of Medicine, Daegu, Republic of Korea; The University of Hong Kong, CHINA

## Abstract

Tumor-associated macrophages (TAMs) are involved in tumor progression by promoting epithelial-mesenchymal transition (EMT), tumor cell invasion, migration and angiogenesis. However, in breast cancer, the clinical relevance of the TAM infiltration according to distinct histologic locations (intratumoral vs. stromal) and hormone receptor status is unclear. We investigated the significance of the levels of TAM infiltration in distinct histologic locations in invasive breast cancer. We also examined the relationship of the TAM levels with the clinicopathologic features of tumors, expression of EMT markers, and clinical outcomes. Finally, we analyzed the prognostic value of TAM levels according to hormone receptor status. High levels of infiltration of intratumoral, stromal and total TAMs were associated with high histologic grade, p53 overexpression, high Ki-67 proliferation index and negative hormone receptor status. Infiltration of TAMs was also correlated with overexpression of vimentin, smooth muscle actin and alteration of β-catenin. Overall, a high level of infiltration of intratumoral TAMs was associated with poor disease-free survival, and was found to be an independent prognostic factor. In subgroup analyses by hormone receptor status, a high level of infiltration of intratumoral TAM was an independent prognostic factor in the hormone receptor-positive subgroup, but not in the hormone-receptor negative subgroup. Our findings suggest that intratumoral TAMs play an important role in tumor progression in breast cancer, especially in the hormone receptor-positive group, and the level of TAM infiltration may be used as a prognostic factor and even a therapeutic target in breast cancer.

## Introduction

The tumor microenvironment contains a diverse leukocyte population, including neutrophils, eosinophils, dendritic cells, macrophages, mast cells and lymphocytes [1]. Macrophages are a major component of the leukocyte population, and are among the most important regulators of tumorigenesis [2]. They have the potential to differentiate into either M1- or M2-polarized macrophages, which have opposing effects on tumor progression. Classically-activated M1 macrophages release pro-inflammatory cytokines together with toxic intermediates and activate a type 1 T-cell response that has a cytotoxic effect on tumor cells, whereas M2 macrophages produce proteolytic enzymes, suppress immune activity and contribute to hypoxia-induced angiogenesis, thereby promoting tumor cell proliferation and migration [3–5]. It has been suggested that tumor-associated macrophages (TAMs) represent a unique and distinct M2-skewed myeloid population [6–8]. In mammary tumors, TAMs were reported to promote cell invasion and the production of colony-stimulating factor-1 (CSF-1) by releasing epidermal growth factor via a paracrine loop [9]. In an animal model, lung metastasis of mammary tumors was delayed in transgenic mice harboring a recessive null mutation in the CSF-1 gene, while transgenic expression of CSF-1 accelerated the progression and metastasis of mammary gland tumors [10]. Therefore, TAMs have been regarded as important mediators of tumor progression in breast cancer.

Epithelial-mesenchymal transition (EMT) is a fundamental process in tumor invasion and metastasis, by which tumor cells lose epithelial properties and acquire mesenchymal characteristics [11]. Increasing evidence suggests that TAMs promote EMT in tumor cells. Singh et al. showed that pro-inflammatory cytokines secreted by macrophages induced the secretion of transforming growth factor-β (TGF-β), which increased the expression of cAMP response element binding protein, thereby activating an EMT response and resulting in increased migration of a non-invasive MCF-7 breast cancer cell line [12]. In addition, it was reported that M2-polarized TAMs promoted EMT in pancreatic cancer cells partially through toll-like receptor 4/ IL-10 signaling [13]. However, there have been few studies of the correlation between TAM infiltration and the expression of EMT markers in human tissue samples, including breast cancers.

Previous studies demonstrated that TAM infiltration is associated with poor clinical outcomes in breast cancer [14–18]. In multiple studies, a high level of infiltration of TAMs was associated with negative hormone receptor status [16–18]. Because hormone receptor-negative breast cancers, including the HER2+ and triple-negative subtypes, are enriched in TAMs and have poor clinical outcomes [19, 20], the relative prognostic significance of elevated TAM infiltration in tumors of differing hormone receptor status needs to be evaluated. Moreover, few studies have focused on the histologic location of TAMs in relation to their clinicopathologic and prognostic significance [17, 18, 21], and thus, understanding of the clinical relevance of the histologic location of TAMs is still lacking and needs to be validated by standardized methods. Therefore, in the present study, we evaluated the significance of TAM levels in different histologic locations (intratumoral vs. stromal) in invasive breast cancer, and the relationship of TAM infiltration with the clinicopathologic features of tumors, expression of EMT markers, and clinical outcomes. We also analyzed the prognostic value of TAM levels according to hormone receptor status.

## Materials and Methods

### Ethics statement

This study was approved by the institutional review board of Seoul National University Bundang Hospital (protocol # B-0909/083-002). The requirements for informed consent from participants were waived by the institutional review board as all the specimen were previously collected for pathologic examination after surgery and all the data were analyzed anonymously.

### Tissue samples

In this study, we used two different sets of breast cancer samples using tissue microarrays. The first set included two hundred-and-seventy-six consecutive cases resected from May 2003 to December 2006, which were used in a previous study [22]. This set was used to evaluate the relationship of TAM infiltration with the clinicopathologic features of tumors, expression of EMT markers, and clinical outcomes. The second set of 175 cases of hormone receptor-negative breast cancer, which were composed of 79 cases from the first set and 96 cases resected from June 2005 to December 2011, was used to assess the prognostic significance of TAM infiltration in hormone receptor-negative breast cancers. Cases receiving preoperative chemotherapy or with evidence of initial metastasis were excluded. The baseline characteristics of the two different sets are summarized in S1 Table.

### Immunohistochemical staining

Immunohistochemical staining was performed on the tissue microarrays. Four μm thick tissue sections were cut, dried, deparaffinized, and rehydrated following standard procedures. The sections were subjected to heat-induced antigen retrieval. Immunohistochemical staining was performed with anti-CD68 antibody (1:300, clone PG-M1, Dako, Carpinteria, CA) with an UltraView detection kit (Ventana Medical Systems, Tucson, AZ) in a BenchMark XT autostainer (Ventana Medical Systems).

Expression of standard biomarkers including estrogen receptor (ER), progesterone receptor (PR), HER2, p53, Ki-67, cytokeratin 5/6, and EGFR and EMT markers (vimentin, smooth muscle actin, osteonectin, N-cadherin, E-cadherin and β-catenin) had been assessed previously [22, 23], and the results were used in this study with the same cut-off values.

### Definition of breast cancer subtypes

According to the St. Gallen Expert Consensus [24], breast cancer subtypes were categorized as: luminal A subtype (ER+ and/or PR+, HER2-, Ki-67<14%), luminal B subtype (ER+ and/or PR+, Ki-67≥14%; ER+ and/or PR+, HER2+), HER2+ subtype (ER-, PR-, and HER2+) and triple-negative subtype (ER-, PR-, and HER2-). For ER and PR, 1% or greater positive staining was defined as positive. For HER2, identification of gene amplification by fluorescence in situ hybridization was considered positive.

### Quantification of tumor-associated macrophages

The tissue microarrays used in this study were 2 mm in diameter, made in triplicate. All tissue microarray slides stained with CD68 were scanned to identify areas with the highest levels of TAM infiltration. For each case, three hot spots in a high-power field (40x objective) were selected for counting TAMs. Each region was photographed and imported into UTHSCSA Image Tool software (Version 3.0, Department of Dental Diagnostic Science at The University of Texas Health Science Center, San Antonio, TX) for counting TAMs. The previously used criteria for localization of tumor infiltrating lymphocytes were applied for distinguishing intratumoral and stromal TAMs [25, 26]. Intratumoral TAMs were defined as macrophages within tumor cell nests and in direct contact with tumor cells, and stromal TAMs were defined as macrophages infiltrated in the tumor stroma of the invasive carcinoma. After counting, the average numbers of intratumoral, stromal and total TAMs (sum of both types) per high power field were calculated.

### Statistical analysis

All analyses were performed using Statistical Package for the Social Sciences software (version 19.0, SPSS inc, Chicago, IL, USA). The Pearson correlation test was used to evaluate the correlations between levels of infiltration of intratumoral, stromal and total TAMs. The median extent of infiltration in each histologic location was used as the cut-off point for assigning tumors into low and high TAM groups. We investigated the association between TAMs and the clinicopathologic features of tumors using the chi-square or Fisher’s exact test. The number of Intratumoral, stromal and total TAMs was also compared by one-way analysis of variance (ANOVA) and the Turkey post hoc test according to breast cancer subtype. A receiver operating characteristic (ROC) curve analysis was performed to identify the most appropriate cut-off values for TAMs that maximized the sum of sensitivity and specificity in predicting clinical outcomes. Survival curves were estimated using the Kaplan–Meier method and compared using the log-rank test. Covariates that were statistically significant in a univariate model were included in a multivariate analysis using the Cox proportional-hazards regression model. Hazard ratios and their 95% confidence intervals were estimated for all factors. P-values <0.05 were considered statistically significant. All p-values were two-sided.

## Results

### Infiltration of TAMs and their association with clinicopathologic features of tumors

**Fig 1:**
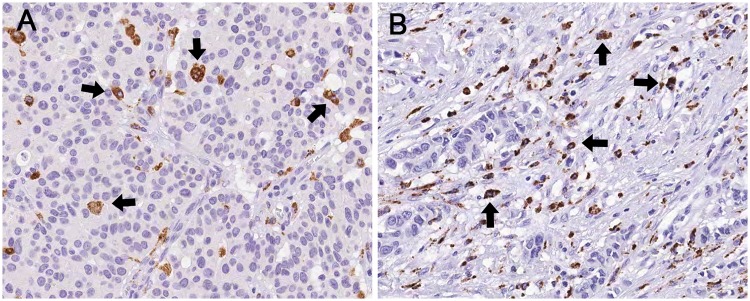
Infiltration of CD68+ tumor-associated macrophages (TAMs) in distinct histologic location. (A) CD68+ TAMs are noted in the intratumoral compartment (arrow). (B) CD68+ TAMs are predominantly found in the stromal compartment (arrow). Original magnification, x400.

**Fig 2:**
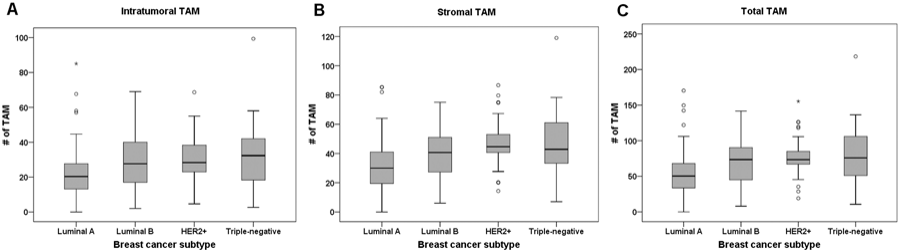
Infiltration of CD68+ tumor-associated macrophages (TAMs) according to breast cancer subtype. The levels of intratumoral (A), stromal (B), and total (C) TAMs are different according to breast cancer subtype. They are significantly lower in luminal A subtype than in luminal B, HER2+ and triple-negative subtypes. The box shows the first to third quartiles of TAMs infiltration levels, the horizontal line inside the box represents the median, the whiskers extend to minimum and maximum values within 1.5 times the interquartile range (IQR) from the first and third quartiles. Outliers are represented by small circles and, extreme values (more than 3 times IQR), by asterisks.

First, we measured the extent of intratumoral and stromal infiltration of TAMs in each tumor in the first set (Fig 1). TAM infiltration levels were variable. The median numbers of intratumoral and stromal TAMs per high-power field were 24.2 (interquartile range: 15.6 to 35.3) and 35.3 (interquartile range: 24.2 to 48.0), respectively. The levels of infiltration of intratumoral, stromal, and total TAMs were highly correlated (intratumoral vs. stromal TAM, r = 0.912, *p*<0.001; intratumoral vs. total TAM, r = 0.972, *p*<0.001; stromal vs. total TAM, r = 0.983, *p*<0.001). The relationships between levels of TAM infiltration (low vs. high) and various clinicopathologic features of the tumors are shown in Table 1. High levels of infiltration of intratumoral, stromal and total TAMs were associated with high histologic grade, pushing border, p53 overexpression, high Ki-67 proliferation index, ER negativity and PR negativity. HER2 status was also positively correlated with stromal and total TAMs, and with intratumoral TAMs (with marginal significance) (*p* = 0.030, *p* = 0.008, *p* = 0.062, respectively). The extent of infiltration of TAMs also differed by breast cancer subtype (*p*<0.001 in all compartments, Chi-square test and one-way ANOVA test), being significantly lower in luminal A subtype than in luminal B, HER2+ and triple-negative subtypes (intratumoral, *p* = 0.008, *p* = 0.006, *p*<0.001; stromal, *p* = 0.005, *p*<0.001, *p*<0.001; total, *p* = 0.005, *p*<0.001, *p*<0.001, respectively, Turkey post hoc test; Fig 2).

**Table 1:** Association of TAMs with the clinicopathologic characteristics of tumors.

Clinicopathologic Characteristic	Intratumoral TAMs		*p* value	Stromal TAMs		*p* value	Total TAMs		*p* value
	Low	High		Low	High		Low	High	
	N (%)	N (%)		N (%)	N (%)		N (%)	N (%)	
Age			0.117			0.630			0.402
<50	66 (47.8)	80 (58.0)		70 (51.1)	76 (54.7)		70 (50.4)	76 (55.5)	
≥50	72 (52.2)	58 (42.0)		67 (48.9)	63 (45.3)		69 (49.6)	61 (44.5)	
T stage			0.749			0.335			0.215
T1-T2	134 (97.1)	132 (95.7)		134 (97.8)	132 (95.0)		136 (97.8)	130 (94.9)	
T3-T4	4 (2.9)	6 (4.3)		3 (2.2)	7 (5.0)		3 (2.2)	7 (5.1)	
N stage			0.718			1.000			0.810
N0	74 (53.6)	70 (50.7)		71 (51.8)	73 (52.5)		74 (53.2)	70 (51.1)	
N1-N3	64 (46.4)	68 (49.3)		66 (48.2)	66 (47.5)		65 (46.8)	67 (48.9)	
Histologic grade			<0.001			<0.001			<0.001
I & II	100 (72.5)	55 (39.9)		103 (75.2)	52 (37.4)		104 (74.8)	51 (37.2)	
III	38 (27.5)	83 (60.1)		34 (24.8)	87 (62.6)		35 (25.2)	86 (62.8)	
Lymphovascular invasion			0.904			1.000			0.809
Absent	76 (55.1)	78 (56.5)		76 (55.5)	78 (56.1)		79 (56.8)	75 (54.7)	
Present	62 (44.9)	60 (43.5)		61 (44.5)	61 (43.9)		60 (43.2)	62 (45.3)	
Tumor border			0.001			0.003			0.004
Pushing	29 (21.0)	55 (39.9)		30 (21.9)	54 (38.8)		31 (22.3)	53 (38.7)	
Infiltrative	109 (79.0)	83 (60.1)		107 (78.1)	85 (61.2)		108 (77.7)	84 (61.3)	
P53 overexpression			0.004			0.001			0.001
Absent	117 (84.8)	96 (69.6)		118 (86.1)	95 (68.3)		119 (85.6)	94 (68.6)	
Present	21 (15.2)	42 (30.4)		19 (13.9)	44 (31.7)		20 (14.4)	43 (31.4)	
Ki-67			<0.001			<0.001			<0.001
<20%	99 (71.7)	64 (46.4)		103 (75.2)	60 (43.2)		104 (74.8)	59 (43.1)	
≥20%	39 (28.3)	74 (53.6)		34 (24.8)	79 (56.8)		35 (25.2)	78 (56.9)	
ER			<0.001			<0.001			<0.001
Negative	25 (18.1)	59 (42.8)		23 (16.8)	61 (43.9)		23 (16.5)	61 (44.5)	
Positive	113 (81.9)	79 (57.2)		114 (83.2)	78 (56.1)		116 (83.5)	76 (55.5)	
PR			0.001			0.001			<0.001
Negative	45 (32.6)	73 (52.9)		45 (32.8)	73 (52.5)		44 (31.7)	74 (54.0)	
Positive	93 (67.4)	65 (47.1)		92 (67.2)	66 (47.5)		95 (68.3)	63 (46.0)	
HER2			0.062			0.030			0.008
Negative	119 (86.2)	106 (76.8)		119 (86.9)	106 (76.3)		122 (87.8)	103 (75.2)	
Positive	19 (13.8)	32 (23.2)		18 (13.1)	33 (23.7)		17 (12.2)	34 (24.8)	
Subtype			<0.001			<0.001			<0.001
Luminal A	86 (62.3)	49 (35.5)		89 (65.0)	46 (33.1)		90 (64.7)	45 (32.8)	
Luminal B	27 (19.6)	35 (25.4)		25 (18.2)	37 (26.6)		26 (18.7)	36 (26.3)	
HER2+	8 (5.8)	21 (15.2)		7 (5.1)	22 (15.8)		6 (4.3)	23 (16.8)	
Triple-negative	17 (12.3)	33 (23.9)		16 (11.7)	34 (24.5)		17 (12.2)	33 (24.1)	

*P* values were calculated by the chi-square or Fisher’s exact test.

TAMs, tumor-associated macrophages; ER, estrogen receptor; PR, progesterone receptor; HER2, human epidermal growth factor receptor 2

In a subgroup analysis according to hormone receptor status, high levels of infiltration of TAMs were associated with high histologic grade and high Ki-67 index in both hormone receptor-positive and hormone receptor-negative groups (S2 and S3 Tables).

### Association of TAMs with expression of EMT markers

**Fig 3:**
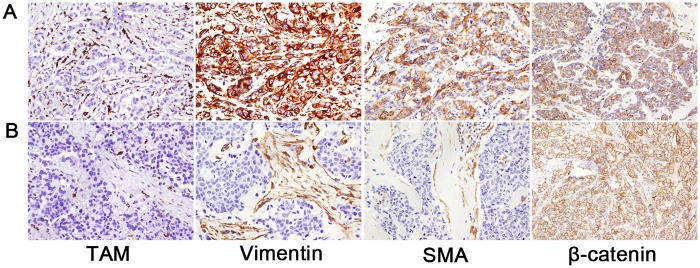
Representative examples showing association of tumor-associated macrophage (TAM) infiltration with expression of epithelial-mesenchymal transition markers. (A) A case with high levels of TAM infiltration shows vimentin and smooth muscle actin (SMA) expression, and alteration of β-catenin. (B) On the contrary, another case with low levels of TAM infiltration does not show vimentin and SMA expression, but reveals intact membranous expression of β-catenin. Original magnification, x200.

The extent of infiltration of TAMs in all histological locations was related to the expression of EMT markers, including vimentin, smooth muscle actin (SMA) and β-catenin (Table 2; Fig 3). In the hormone receptor-positive group, only β-catenin alteration was associated with high infiltration of total TAMs (*p* = 0.046) (S4 Table). In the hormone receptor-negative group, β-catenin alteration was also correlated with elevated infiltration of intratumoral and stromal TAMs (*p* = 0.021, *p* = 0.017, respectively) (S5 Table).

**Table 2:** Association of TAMs with expression of epithelial-mesenchymal transition markers.

Marker	Intratumoral TAMs		*p* value	Stromal TAMs		*p* value	Total TAMs		*p* value
	Low	High		Low	High		Low	High	
	N (%)	N (%)		N (%)	N (%)		N (%)	N (%)	
Vimentin			0.011			0.017			0.029
<10%	112 (91.8)	102 (80.3)		110 (91.7)	104 (80.6)		111 (91.0)	103 (81.1)	
≥10%	10 (8.2)	25 (19.7)		10 (8.3)	25 (19.4)		11 (9.0)	24 (18.9)	
SMA			0.036			0.004			0.036
<1%	121 (99.2)	119 (93.7)		120 (100.0)	120 (93.0)		121 (99.2)	119 (93.7)	
≥1%	1 (0.8)	8 (6.3)		0 (0)	9 (7.0)		1 (0.8)	8 (6.3)	
Osteonectin			0.085			0.226			0.085
<1%	117 (95.9)	114 (89.8)		114 (95.0)	117 (90.7)		117 (95.9)	114 (89.8)	
≥1%	5 (4.1)	13 (10.2)		6 (5.0)	12 (9.3)		5 (4.1)	13 (10.2)	
E-cadherin loss			0.060			0.141			0.179
<10%	89 (73.0)	78 (61.4)		86 (71.7)	81 (62.8)		87 (71.3)	80 (63.0)	
≥10%	33 (27.0)	49 (38.6)		34 (28.3)	48 (37.2)		35 (28.7)	47 (37.0)	
N-cadherin			0.831			0.668			0.670
<10%	111 (91.0)	114 (89.8)		107 (89.2)	118 (91.5)		109 (89.3)	116 (91.3)	
≥10%	11 (9.0)	13 (10.2)		13 (10.8)	11 (8.5)		13 (10.7)	11 (8.7)	
β-catenin alteration			<0.001			<0.001			<0.001
<10%	113 (92.6)	91 (71.7)		112 (93.3)	92 (71.3)		112 (91.8)	92 (72.4)	
≥10%	9 (7.4)	36 (28.3)		8 (6.7)	37 (28.7)		10 (8.2)	35 (27.6)	

*P* values were calculated by the chi-square or Fisher’s exact test.

TAMs, tumor-associated macrophages; SMA, smooth muscle actin

As the expression of EMT markers is predominantly found in triple-negative breast cancers [23], we also evaluated the association of TAM infiltration levels with expression of EMT markers in triple-negative breast cancers and non-triple-negative breast cancers. In triple-negative breast cancers, β-catenin alteration was associated with high levels of infiltration of TAMs in all compartments (intratumoral, *p* = 0.023; stromal, *p* = 0.001; total, *p* = 0.023), and SMA expression was associated with a high level of infiltration of stromal TAMs (*p* = 0.042) (S6 Table). In non-triple-negative breast cancers, β-catenin alteration also showed a positive correlation with infiltration levels of intratumoral and total TAMs (*p* = 0.010, *p* = 0.035, respectively), and vimentin expression was positively correlated with infiltration levels of intratumoral TAM (*p* = 0.048) (S7 Table).

### TAM infiltration and clinical outcomes

**Fig 4:**
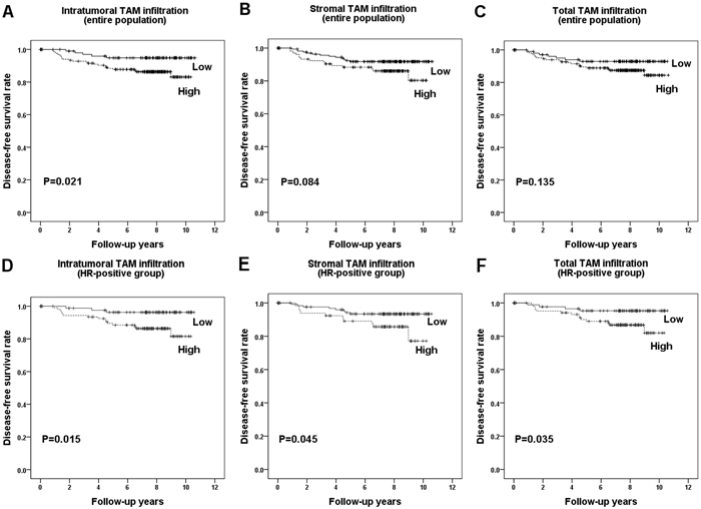
Disease-free survival according to the degree of tumor-associated macrophage (TAM) infiltration in each compartment. High levels of infiltration of intratumoral (A), but not stromal (B) and total (C) TAM, are associated with poor disease-free survival of the entire population of patients. However, in the hormone receptor (HR)-positive group, high levels of infiltration of intratumoral (D), stromal (E) and total (F) TAMs are each associated with decreased disease-free survival.

We also investigated the prognostic significance of TAM infiltration levels according to the different histologic locations and hormone receptor status in the first set. Most patients were treated by the standard practice guidelines and have been followed regularly after surgery. The median follow-up period was 7.7 years at the time of analysis (range, 0.1~10.6 years). There were 6 (2.2%) loco-regional recurrences and twenty-two (8.0%) distant metastases as first events. In the entire tumor sample, high infiltration levels of intratumoral TAMs were associated with decreased disease-free survival (*p* = 0.021; Fig 4A; Table 3). Elevated stromal and total TAM levels tended to be associated with poor disease-free survival (*p* = 0.084, *p* = 0.135, respectively; Fig 4B and 4C). In a multivariate analysis including T stage, N stage, lymphovascular invasion and intratumoral TAMs, high infiltration of intratumoral TAMs (hazard ratio, 2.810; 95% confidence interval, 1.064–7.416; *p* = 0.037) remained an independent prognostic indicator of poor disease-free survival.

**Table 3:** Univariate analysis of disease-free survival.

Group	Variable	*p* value
Entire population	T stage (1–2 vs. 3–4)	0.028
	N stage (0 vs. 1–3)	0.029
	Histologic grade (I-II vs. III)	0.566
	Lymphovascular invasion (absent vs. present)	0.010
	P53 overexpression (absent vs. present)	0.807
	Ki-67 index (<20% vs. ≥20%)	0.552
	ER (negative vs. positive)	0.493
	PR (negative vs. positive)	0.222
	HER2 (negative vs. positive)	0.937
	Intratumoral TAMs (low vs. high)	0.021
	Stromal TAMs (low vs. high)	0.084
	Total TAMs (low vs. high)	0.135
Hormone receptor-positive group	T stage (1–2 vs. 3–4)	0.409
	N stage (0 vs. 1–3)	0.005
	Histologic grade (I-II vs. III)	0.874
	Lymphovascular invasion (absent vs. present)	0.079
	P53 overexpression (absent vs. present)	0.300
	Ki-67 index (<20% vs. ≥20%)	0.359
	HER2 (negative vs. positive)	0.974
	Intratumoral TAMs (low vs. high)	0.015
	Stromal TAMs (low vs. high)	0.045
	Total TAMs (low vs. high)	0.035

*P* values were calculated by the log-rank test.

ER, estrogen receptor; PR, progesterone receptor; HER2, human epidermal growth factor receptor 2

TAMs, tumor-associated macrophages

In the hormone receptor-positive group, high levels of infiltration of intratumoral, stromal and total TAMs were associated with poor disease-free survival (*p* = 0.015, *p* = 0.045, *p* = 0.035, respectively; Table 3; Fig 4D–4F). Since they were highly correlated with each other (r≥0.910, *p<*0.001), multivariate analyses were conducted in different models. In multivariate analyses including N stage and infiltration levels of TAMs in each compartment, N stage and intratumoral TAM levels remained independent predictive factors for poor disease-free survival (Table 4).

**Table 4:** Multivariate analysis by the Cox proportional hazard model in the hormone receptor-positive group.

Model	Variable	Hazard ratio	95% confidence interval	*p* value
A	N stage (0 vs. 1–3)	4.510	1.303–15.605	0.017
	Intratumoral TAMs (low vs. high)	3.772	1.074–12.900	0.038
B	N stage (0 vs. 1–3)	4.687	1.354–16.225	0.015
	Stromal TAMs (low vs. high)	2.186	0.865–5.526	0.098
C	N stage (0 vs. 1–3)	4.737	1.370–16.374	0.014
	Total TAMs (low vs. high)	2.923	0.960–8.903	0.059

TAMs, Tumor-associated macrophages

In the hormone receptor-negative group, the optimal cut-off point could not be obtained by ROC curve analysis (area under curve < 0.5). When using the median as a cutoff value, TAM infiltration levels in none of the compartments were associated with prognosis. However, as the number of the hormone receptor-negative cases was limited in the first set, we extended the study and re-evaluated the prognostic significance of TAMs using the second set composed of 175 hormone receptor-negative breast cancers. Median follow-up period was 5.2 years (range, 0.1~10.6 years), and there were 7 (4.0%) loco-regional recurrences and 16 (9.1%) distant metastases as the first event. In ROC curve analysis, optimal cut-off values were selected for intratumoral and total TAMs, but could not be obtained for stromal TAMs. In survival analysis, intratumoral and total TAM infiltration levels were not associated with disease-free survival of the patients (intratumoral, *p* = 0.143; total, *p* = 0.286, respectively; S1 Fig).

## Discussion

In this study we showed that high levels of infiltration of TAMs in all histological locations were associated with aggressive features of breast cancer and EMT. In addition, high levels of intratumoral TAM infiltration were revealed as an independent poor prognostic factor in the entire patient population with breast cancer, and in the hormone receptor-positive group in particular.

TAMs have various functions according to their microenvironment and histologic location [27]. However, However, little attention has been paid to the significance of the histological location of TAMs in breast cancers except for a few studies [17, 18, 21]. It was demonstrated that tumor stromal TAM density had more prognostic value than tumor nest or intratumoral TAMs [17, 18]. With regard to the clinicopathologic features of tumors, Ch’ng et al. showed that only elevated tumor stromal macrophages were associated with aggressive histologic features of tumors [21]. Medrek et al. also reported that only tumor stromal CD163+ and CD68+ TAMs, but not tumor nest TAMs, were positively correlated with large tumor size and high grades, and inversely correlated with luminal A subtype [17]. However, in this study, high levels of TAM in all histological locations showed a close relationship with aggressive characteristics of tumor, and high levels of intratumoral TAMs were found to be an independent poor prognostic factor. These discrepancies may be explained by several factors, such as the use of different methods to evaluate TAM levels (direct counting vs. semi-quantitative scoring; counting of overall tumor area vs. counting of hot spots), different follow-up periods, and differences in sample size. Further studies using standardized methods will therefore be needed to verify the significance of the TAM levels in distinct histologic locations.

The reason why intratumoral TAM levels are relevant to clinical outcome is unclear. However, Leek et al. demonstrated that hypoxia-associated tumor necrosis attracts macrophages into tumors, and these then contribute to angiogenesis and a poor prognosis [28]. Ch’ng *et al*. proposed that tumor stromal TAMs influence tubular architecture and, eventually, tumor grade, whereas tumor nest TAMs have a closer relationship with hypoxia-induced angiogenesis, indicating that the TAMs have different effects on tumor progression according to their histologic location [21]. It may be possible that the central area in the tumor nest becomes more hypoxic as tumors outgrow their blood supply, and then, intratumoral TAMs which are recruited by hypoxia-induced tumor necrosis become more active in angiogenesis and tumor progression. Therefore, intratumoral TAMs, as well as their stromal counterpart, seems to play an important role in tumor progression.

We showed that only in the hormone receptor-positive group did TAM levels have prognostic significance. Similarly, Medrek et al. evaluated the prognostic value of CD163+ macrophages in tumor stroma in the luminal A and triple-negative subtypes, and found that dense infiltration of CD163+ macrophages was associated with poor overall survival in luminal A subtype, but not in triple-negative subtype [17]. However, they only evaluated tumor stromal TAMs according to breast cancer subtype. In this study, we found that TAMs in all histologic locations had prognostic value, and especially, intratumoral TAM was revealed as an independent prognostic factor in hormone-receptor positive group. Although TAM infiltration in all compartments was correlated with hormone receptor negativity, it did not confer a prognostic significance in the hormone receptor-negative group. As hormone receptor-negative breast cancers are generally enriched with TAMs, and have an unfavorable prognosis compared to hormone-receptor positive breast cancers, TAM levels may have less effect on clinical outcomes in this group. However, larger studies are still necessary to evaluate the clinical significance of TAMs in hormone receptor-negative breast cancers.

In this study we also investigated the relationship of TAM levels with EMT, which is a critical process in tumor progression and metastasis. Intratumoral, stromal and total TAM levels were correlated with the overexpression of mesenchymal markers, including vimentin and SMA, and with β-catenin alteration. Bond et al. also identified a positive correlation between intratumoral macrophage densities, EMT markers, intratumoral TGF-β levels and tumor grade in non-small cell lung cancer [29]. They demonstrated a positive correlation between TAM densities and mesenchymal marker expression by gene expression analysis, and observed that TAMs clustered with EMT phenotypes in tumor cells, suggesting that TAMs affect tumor progression by inducing EMT. It was reported that TAMs secreted more TGF-β1 than other phenotypes of macrophages and promoted cancer stem cell-like properties via TGF-β1—induced EMT in hepatocellular carcinoma [30]. Su et al showed that mesenchymal-like breast cancer cells activated macrophages to a TAM-like phenotype by GM-CSF, and chemokine (C-C motif) ligand 18 (CCL18) from TAMs induced EMT of breast cancer cells, forming a positive feedback loop. Moreover, they also demonstrated that high GM-CSF expression in breast cancer samples was associated with more CCL18 (+) macrophages, cancer cell EMT, enhanced metastasis, and reduced patient survival, implying that interaction between cancer cells with EMT and TAMs is important in breast cancer metastasis [31]. Corresponding with their results, in our study, TAMs in all compartments showed a strong association with expression of EMT markers, suggesting that TAMs contribute to EMT and thus, tumor progression and metastasis.

Of the EMT marker used in this study, β-catenin is noteworthy. Its alteration was associated with high levels of TAM infiltration in both triple-negative and non-triple-negative breast cancers (also in both hormone receptor-negative and hormone receptor-positive breast cancers). The reason why only β-catenin, but not other EMT markers, showed a positive correlation with infiltration levels of TAM in subgroup analysis is not clear. However, a recent study revealed that TAMs inhibited the canonical Wnt pathway and activated the non-canonical Wnt pathway in canine mammary tumor cells, suggesting that TAMs mediate a “switch” between canonical and non-canonical Wnt signaling pathways [32]. The canonical Wnt pathway involves β-catenin, and thus, close association of TAM infiltration with β-catenin alteration in this study may be explained by the inhibitory action of TAMs on canonical Wnt pathway.

In summary, we showed that dense infiltration of TAMs in all histologic compartments was associated with aggressive features of breast cancer and EMT. More importantly, dense infiltration of intratumoral TAMs appears to be an independent poor prognostic factor in breast cancer patients in general, and in hormone receptor-positive patients in particular. We therefore propose that intratumoral TAMs as well as stromal TAMs play a critical role in tumor progression in breast cancer, especially in the hormone receptor-positive subgroup, and they can be used as a prognostic factor and a potential therapeutic target in breast cancer.

## Supporting Information

S1 Fig Disease-free survival according to the degree of tumor-associated macrophage (TAM) infiltration in hormone receptor (HR)-negative breast cancers. Levels of infiltration of intratumoral (A) and total (B) TAM, are not associated with disease-free survival of the patients in HR-negative breast cancers.
(TIF)

S1 Table Baseline tumor characteristics. Clinicopathologic characteristics of tumors in the first and second set were presented.
(DOCX)

S2 Table Association of TAMs with the clinicopathologic characteristics of tumors in the hormone receptor-positive group. High levels of infiltration of TAMs were associated with high histologic grade and high Ki-67 index.
(DOCX)

S3 Table Association of TAMs with the clinicopathologic characteristics of tumors in the hormone receptor-negative group. High levels of infiltration of TAMs were associated with high histologic grade, pushing border and high Ki-67 index.
(DOCX)

S4 Table Association of TAMs with expression of epithelial-mesenchymal transition markers in the hormone receptor-positive group. β-catenin alteration was associated with elevated infiltration of total TAMs.
(DOCX)

S5 Table Association of TAMs with expression of epithelial-mesenchymal transition markers in the hormone receptor-negative group. β-catenin alteration was associated with high levels of intratumoral and stromal TAM infiltration.
(DOCX)

S6 Table Association of TAMs with expression of epithelial-mesenchymal transition markers in triple-negative breast cancers. β-catenin alteration showed a positive correlation with infiltration levels of TAMs in all compartments, and SMA expression was positively correlated with infiltration level of stromal TAMs.
(DOCX)

S7 Table Association of TAMs with expression of epithelial-mesenchymal transition markers in non-triple-negative breast cancers. β-catenin alteration was associated with high infiltration of intratumoral and total TAMs, and vimentin expression was associated with high infiltration of intratumoral TAMs.
(DOCX)
