# Supplementary figures and images for: Prognostic Value of Tumor-Associated Macrophages According to Histologic Locations and Hormone Receptor Status in Breast Cancer
Source: PLoS One. 2015 Apr 17;10(4):e0125728. doi: 10.1371/journal.pone.0125728 (PMC4401667; doi:10.1371/journal.pone.0125728)

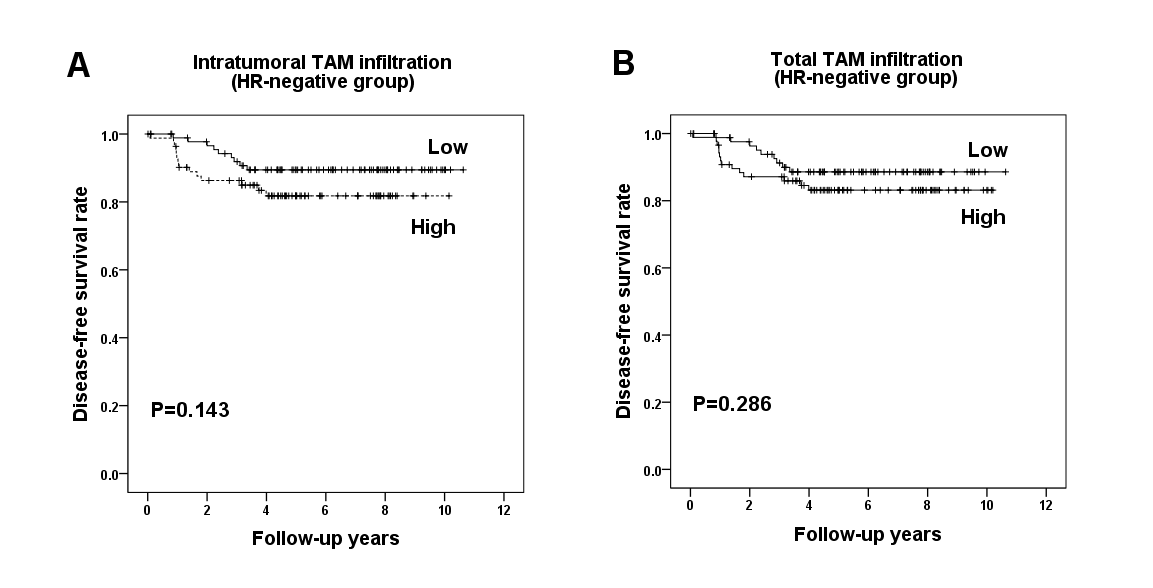

Supplement: S1 Fig — Levels of infiltration of intratumoral (A) and total (B) TAM, are not associated with disease-free survival of the patients in HR-negative breast cancers. (TIF) [file pone.0125728.s001.tif]
