# Supplementary material for: Prognostic Value of Tumor-Associated Macrophages According to Histologic Locations and Hormone Receptor Status in Breast Cancer
Source: PLoS One. 2015 Apr 17;10(4):e0125728. doi: 10.1371/journal.pone.0125728 (PMC4401667; doi:10.1371/journal.pone.0125728)
Supplement: S1 Table — Clinicopathologic characteristics of tumors in the first and second set were presented. (DOCX) [file pone.0125728.s002.docx]

**S1 Table.** Baseline tumor characteristics

| **Characteristics** | **The first set** | **The second set** |
| --- | --- | --- |
|  | **N (%)** | **N (%)** |
| Age, yrs. |  |  |
| Mean ± SD | 50.54 (±11.43) | 51.62 (±12.70) |
| Range | 26-87 | 26-87 |
| T stage |  |  |
| T1 | 139 (50.4) | 74 (42.3) |
| T2 | 127 (46.0) | 94 (53.7) |
| T3 | 5 (1.8) | 5 (2.9) |
| T4 | 5 (1.8) | 2 (1.1) |
| N stage |  |  |
| N0 | 144 (52.2) | 119 (68.0) |
| N1 | 79 (28.6) | 31 (17.7) |
| N2 | 30 (10.9) | 13 (7.4) |
| N3 | 23 (8.3) | 12 (6.9) |
| Histologic grade |  |  |
| I | 65 (23.6) | 2 (1.1) |
| II | 90 (32.6) | 17 (9.7) |
| III | 121 (43.8) | 156 (89.1) |
| Lymphovascular invasion |  |  |
| Absent | 154 (55.8) | 106 (60.6) |
| Present | 122 (44.2) | 69 (39.4) |
| Tumor border |  |  |
| Pushing | 84 (30.4) | 97 (55.4) |
| Infiltrative | 192 (69.6) | 78 (44.6) |
| P53 overexpression |  |  |
| Absent | 213 (77.2) | 74 (42.3) |
| Present | 63 (22.8) | 101 (57.7) |
| Ki-67 |  |  |
| <20% | 163 (59.1) | 27 (15.4) |
| ≥20% | 113 (40.9) | 148 (84.6) |
| ER |  |  |
| Negative | 84 (30.4) | 175 (100.0) |
| Positive | 192 (69.6) | 0 (0) |
| PR |  |  |
| Negative | 118 (42.8) | 175 (100.0) |
| Positive | 158 (57.2) | 0 (0) |
| HER2 |  |  |
| Negative | 225 (81.5) | 120 (68.6) |
| Positive | 51 (18.5) | 55 (31.4) |
| Subtype |  |  |
| Luminal A | 135 (48.9) | 0 (0) |
| Luminal B | 62 (22.5) | 0 (0) |
| HER2+ | 29 (10.5) | 55 (31.4) |
| Triple-negative | 50 (18.1) | 120 (68.6) |

SD, standard deviation; ER, estrogen receptor; PR, progesterone receptor; HER2, human epidermal growth factor receptor 2
