# Supplementary material for: Prognostic Value of Tumor-Associated Macrophages According to Histologic Locations and Hormone Receptor Status in Breast Cancer
Source: PLoS One. 2015 Apr 17;10(4):e0125728. doi: 10.1371/journal.pone.0125728 (PMC4401667; doi:10.1371/journal.pone.0125728)
Supplement: S2 Table — High levels of infiltration of TAMs were associated with high histologic grade and high Ki-67 index. (DOCX) [file pone.0125728.s003.docx]

**S2 Table.** Association of TAMs with the clinicopathologic characteristics of tumors in the hormone receptor-positive group

| **Clinicopathologic Characteristics** | **Intratumoral TAMs** | | ***p* value** | **Stromal TAMs** | | ***p* value** | **Total TAMs** | | ***p* value** |
| --- | --- | --- | --- | --- | --- | --- | --- | --- | --- |
|  | **Low** | **High** |  | **Low** | **High** |  | **Low** | **High** |  |
|  | **N (%)** | **N (%)** |  | **N (%)** | **N (%)** |  | **N (%)** | **N (%)** |  |
| Age |  |  | 0.083 |  |  | 0.192 |  |  | 0.150 |
| <50 | 55 (48.7) | 52 (61.9) |  | 57 (50.0) | 50 (60.2) |  | 58 (50.0) | 49 (60.5) |  |
| ≥50 | 58 (51.3) | 32 (38.1) |  | 57 (50.0) | 33 (39.8) |  | 58 (50.0) | 32 (39.5) |  |
| T stage |  |  | 0.653 |  |  | 0.164 |  |  | 0.161 |
| T1-T2 | 111 (98.2) | 81 (96.4) |  | 113 (99.1) | 79 (95.2) |  | 115 (99.1) | 77 (95.1) |  |
| T3-T4 | 2 (1.8) | 3 (3.6) |  | 1 (0.9) | 4 (4.8) |  | 1 (0.9) | 4 (4.9) |  |
| N stage |  |  | 0.199 |  |  | 0.567 |  |  | 0.250 |
| N0 | 59 (52.2) | 36 (42.9) |  | 57 (50.0) | 38 (45.8) |  | 60 (51.7) | 35 (43.2) |  |
| N1-N3 | 54 (47.8) | 48 (57.1) |  | 57 (50.0) | 45 (54.2) |  | 56 (48.3) | 46 (56.8) |  |
| Histologic grade |  |  | 0.004 |  |  | <0.001 |  |  | 0.001 |
| I & II | 90 (79.6) | 51 (60.7) |  | 93 (81.6) | 48 (57.8) |  | 94 (81.0) | 47 (58.0) |  |
| III | 23 (20.4) | 33 (39.3) |  | 21 (18.4) | 35 (42.2) |  | 22 (19.0) | 34 (42.0) |  |
| Lymphovascular invasion |  |  | 0.774 |  |  | 0.472 |  |  | 0.194 |
| Absent | 62 (54.9) | 44 (52.4) |  | 64 (56.1) | 42 (50.6) |  | 67 (57.8) | 39 (48.1) |  |
| Present | 51 (45.1) | 40 (47.6) |  | 50 (43.9) | 41 (49.4) |  | 49 (42.2) | 42 (51.9) |  |
| Tumor border |  |  | 0.858 |  |  | 1.000 |  |  | 0.859 |
| Pushing | 22 (19.5) | 18 (21.4) |  | 23 (20.2) | 17 (20.5) |  | 23 (19.8) | 17 (21.0) |  |
| Infiltrative | 91 (80.5) | 66 (78.6) |  | 91 (79.8) | 66 (79.5) |  | 93 (80.2) | 64 (79.0) |  |
| P53 overexpression |  |  | 0.465 |  |  | 0.464 |  |  | 0.331 |
| Negative | 104 (92.0) | 74 (88.1) |  | 105 (92.1) | 73 (88.0) |  | 107 (92.2) | 71 (87.7) |  |
| Positive | 9 (8.0) | 10 (11.9) |  | 9 (7.9) | 10 (12.0) |  | 9 (7.8) | 10 (12.3) |  |
| Ki-67 |  |  | 0.068 |  |  | 0.004 |  |  | 0.007 |
| <20% | 91 (80.5) | 58 (69.0) |  | 95 (83.3) | 54 (65.1) |  | 96 (82.8) | 53 (65.4) |  |
| ≥20% | 22 (19.5) | 26 (31.0) |  | 19 (16.7) | 29 (34.9) |  | 20 (17.2) | 28 (34.6) |  |

*P* value was calculated by chi-square test or Fisher’s exact test.

TAMs, tumor-associated macrophages
