# Supplementary material for: Prognostic Value of Tumor-Associated Macrophages According to Histologic Locations and Hormone Receptor Status in Breast Cancer
Source: PLoS One. 2015 Apr 17;10(4):e0125728. doi: 10.1371/journal.pone.0125728 (PMC4401667; doi:10.1371/journal.pone.0125728)
Supplement: S3 Table — High levels of infiltration of TAMs were associated with high histologic grade, pushing border and high Ki-67 index. (DOCX) [file pone.0125728.s004.docx]

**S3 Table.** Association of TAMs with the clinicopathologic characteristics of tumors in the hormone receptor-negative group

| **Clinicopathologic Characteristics** | **Intratumoral TAMs** | | ***p value*** | **Stromal TAMs** | | ***p value*** | **Total TAMs** | | ***p value*** |
| --- | --- | --- | --- | --- | --- | --- | --- | --- | --- |
|  | **Low** | **High** |  | **Low** | **High** |  | **Low** | **High** |  |
|  | **N (%)** | **N (%)** |  | **N (%)** | **N (%)** |  | **N (%)** | **N (%)** |  |
| Age |  |  | 0.630 |  |  | 0.465 |  |  | 0.808 |
| <50 | 11 (44.0) | 28 (51.9) |  | 13 (56.5) | 26 (46.4) |  | 12 (52.2) | 27 (48.2) |  |
| ≥50 | 14 (56.0) | 26 (48.1) |  | 10 (43.5) | 30 (53.6) |  | 11 (47.8) | 29 (51.8) |  |
| T stage |  |  | 0.649 |  |  | 0.625 |  |  | 0.625 |
| T1-T2 | 23 (92.0) | 51 (94.4) |  | 21 (91.3) | 53 (94.6) |  | 21 (91.3) | 53 (94.6) |  |
| T3-T4 | 2 (8.0) | 3 (5.6) |  | 2 (8.7) | 3 (5.4) |  | 2 (8.7) | 3 (5.4) |  |
| N stage |  |  | 0.808 |  |  | 1.000 |  |  | 1.000 |
| N0 | 15 (60.0) | 34 (63.0) |  | 14 (60.9) | 35 (62.5) |  | 14 (60.9) | 35 (62.5) |  |
| N1-N3 | 10 (40.0) | 20 (37.0) |  | 9 (39.1) | 21 (37.5) |  | 9 (39.1) | 21 (37.5) |  |
| Histologic grade |  |  | 0.001 |  |  | <0.001 |  |  | <0.001 |
| I & II | 10 (40.0) | 4 (7.4) |  | 10 (43.5) | 4 (7.1) |  | 10 (43.5) | 4 (7.1) |  |
| III | 15 (60.0) | 50 (92.6) |  | 13 (56.5) | 52 (92.9) |  | 13 (56.5) | 52 (92.9) |  |
| Lymphovascular invasion |  |  | 0.624 |  |  | 0.447 |  |  | 0.447 |
| Absent | 14 (56.0) | 34 (63.0) |  | 12 (52.2) | 36 (64.3) |  | 12 (52.2) | 36 (64.3) |  |
| Present | 11 (44.0) | 20 (37.0) |  | 11 (47.8) | 20 (35.7) |  | 11 (47.8) | 20 (35.7) |  |
| Tumor border |  |  | 0.001 |  |  | 0.006 |  |  | 0.024 |
| Pushing | 7 (28.0) | 37 (68.5) |  | 7 (30.4) | 37 (66.1) |  | 8 (34.8) | 36 (64.3) |  |
| Infiltrative | 18 (72.0) | 17 (31.5) |  | 16 (69.6) | 19 (33.9) |  | 15 (65.2) | 20 (35.7) |  |
| P53 overexpression |  |  | 0.466 |  |  | 0.214 |  |  | 0.457 |
| Negative | 13 (52.0) | 22 (40.7) |  | 13 (56.5) | 22 (39.3) |  | 12 (52.2) | 23 (41.1) |  |
| Positive | 12 (48.0) | 32 (59.3) |  | 10 (43.5) | 34 (60.7) |  | 11 (47.8) | 33 (58.9) |  |
| Ki-67 |  |  | 0.053 |  |  | 0.020 |  |  | 0.020 |
| <20% | 8 (32.0) | 6 (11.1) |  | 8 (34.8) | 6 (10.7) |  | 8 (34.8) | 6 (10.7) |  |
| ≥20% | 17 (68.0) | 48 (88.9) |  | 15 (65.2) | 50 (89.3) |  | 15 (65.2) | 52 (89.3) |  |

*P* value was calculated by chi-square test or Fisher’s exact test

TAMs, tumor-associated macrophages
