# Supplementary material for: Prognostic Value of Tumor-Associated Macrophages According to Histologic Locations and Hormone Receptor Status in Breast Cancer
Source: PLoS One. 2015 Apr 17;10(4):e0125728. doi: 10.1371/journal.pone.0125728 (PMC4401667; doi:10.1371/journal.pone.0125728)
Supplement: S4 Table — β-catenin alteration was associated with elevated infiltration of total TAMs. (DOCX) [file pone.0125728.s005.docx]

**S4 Table.** Association of TAMs with expression of epithelial-mesenchymal transition markers in the hormone receptor-positive group

| **Marker** | **Intratumoral TAMs** | | ***p value*** | **Stromal TAMs** | | ***p value*** | **Total TAMs** | | ***p value*** |
| --- | --- | --- | --- | --- | --- | --- | --- | --- | --- |
|  | **Low** | **High** |  | **Low** | **High** |  | **Low** | **High** |  |
|  | **N (%)** | **N (%)** |  | **N (%)** | **N (%)** |  | **N (%)** | **N (%)** |  |
| Vimentin |  |  | 0.405 |  |  | 1.000 |  |  | 0.697 |
| <10% | 98 (98.0) | 72 (94.7) |  | 97 (97.0) | 73 (96.1) |  | 99 (97.1) | 71 (95.9) |  |
| ≥10% | 2 (2.0) | 4 (5.3) |  | 3 (3.0) | 3 (3.9) |  | 3 (2.9) | 3 (4.1) |  |
| SMA |  |  | * |  |  | * |  |  | * |
| <1% | 100 (100.0) | 76 (100.0) |  | 100 (100.0) | 76 (100.0) |  | 102 (100.0) | 74 (100.0) |  |
| ≥1% | 0 (0) | 0 (0) |  | 0 (0) | 0 (0) |  | 0 (0) | 0 (0) |  |
| Osteonectin |  |  | 0.467 |  |  | 0.467 |  |  | 0.456 |
| <1% | 97 (97.0) | 72 (94.7) |  | 97 (97.0) | 72 (94.7) |  | 99 (97.1) | 70 (94.6) |  |
| ≥1% | 3 (3.0) | 4 (5.3) |  | 3 (3.0) | 4 (5.3) |  | 3 (2.9) | 4 (5.4) |  |
| E-cadherin loss |  |  | 1.000 |  |  | 0.584 |  |  | 0.714 |
| <10% | 78 (78.0) | 59 (77.6) |  | 76 (76.0) | 61 (80.3) |  | 78 (76.5) | 59 (79.7) |  |
| ≥10% | 22 (22.0) | 17 (22.4) |  | 24 (24.0) | 15 (19.7) |  | 24 (23.5) | 15 (20.3) |  |
| N-cadherin |  |  | 1.000 |  |  | 0.759 |  |  | 0.763 |
| <10% | 94 (94.0) | 71 (93.4) |  | 93 (93.0) | 72 (94.7) |  | 95 (93.1) | 70 (94.6) |  |
| ≥10% | 6 (6.0) | 5 (6.6) |  | 7 (7.0) | 4 (5.3) |  | 7 (6.9) | 4 (5.4) |  |
| β-catenin alteration |  |  | 0.078 |  |  | 0.078 |  |  | 0.046 |
| <10% | 96 (96.0) | 67 (88.2) |  | 96 (96.0) | 67 (88.2) |  | 98 (96.1) | 65 (87.8) |  |
| ≥10% | 4 (4.0) | 9 (11.8) |  | 4 (4.0) | 9 (11.8) |  | 4 (3.9) | 9 (12.2) |  |

*P* values were calculated by the chi-square or Fisher’s exact test

TAMs, tumor-associated macrophages; SMA, smooth muscle actin

* could not be calculated
