# Supplementary material for: Prognostic Value of Tumor-Associated Macrophages According to Histologic Locations and Hormone Receptor Status in Breast Cancer
Source: PLoS One. 2015 Apr 17;10(4):e0125728. doi: 10.1371/journal.pone.0125728 (PMC4401667; doi:10.1371/journal.pone.0125728)
Supplement: S5 Table — β-catenin alteration was associated with high levels of intratumoral and stromal TAM infiltration. (DOCX) [file pone.0125728.s006.docx]

**S5 Table.** Association of TAMs with expression of epithelial-mesenchymal transition markers in the hormone receptor-negative group

| **Marker** | **Intratumoral TAMs** | | ***p value*** | **Stromal TAMs** | | ***p value*** | **Total TAMs** | | ***p value*** |
| --- | --- | --- | --- | --- | --- | --- | --- | --- | --- |
|  | **Low** | **High** |  | **Low** | **High** |  | **Low** | **High** |  |
|  | **N (%)** | **N (%)** |  | **N (%)** | **N (%)** |  | **N (%)** | **N (%)** |  |
| Vimentin |  |  | 0.797 |  |  | 0.789 |  |  | 1.000 |
| <10% | 14 (63.6) | 30 (58.8) |  | 13 (65.0) | 31 (58.5) |  | 12 (60.0) | 32 (60.4) |  |
| ≥10% | 8 (36.4) | 21 (41.2) |  | 7 (35.0) | 22 (41.5) |  | 8 (40.0) | 21 (39.6) |  |
| SMA |  |  | 0.262 |  |  | 0.056 |  |  | 0.429 |
| <1% | 21 (95.5) | 43 (84.3) |  | 20 (100.0) | 44 (83.0) |  | 19 (95.0) | 45 (84.9) |  |
| ≥1% | 1 (4.5) | 8 (15.7) |  | 0 (0) | 9 (17.0) |  | 1 (5.0) | 8 (15.1) |  |
| Osteonectin |  |  | 0.486 |  |  | 1.000 |  |  | 0.716 |
| <1% | 20 (90.9) | 42 (82.4) |  | 17 (85.0) | 45 (84.9) |  | 18 (90.0) | 44 (83.0) |  |
| ≥1% | 2 (9.1) | 9 (17.6) |  | 3 (15.0) | 8 (15.1) |  | 2 (10.0) | 9 (17.0) |  |
| E-cadherin loss |  |  | 0.437 |  |  | 0.426 |  |  | 0.791 |
| <10% | 11 (50.0) | 19 (37.3) |  | 10 (50.0) | 20 (37.7) |  | 9 (45.0) | 21 (39.6) |  |
| ≥10% | 11 (50.0) | 32 (62.7) |  | 10 (50.0) | 33 (62.3) |  | 11 (55.0) | 32 (60.4) |  |
| N-cadherin |  |  | 0.514 |  |  | 0.166 |  |  | 0.166 |
| <10% | 17 (77.3) | 43 (84.3) |  | 14 (70.0) | 46 (86.8) |  | 14 (70.0) | 46 (86.8) |  |
| ≥10% | 5 (22.7) | 8 (15.7) |  | 6 (30.0) | 7 (13.2) |  | 6 (30.0) | 7 (13.2) |  |
| β-catenin alteration |  |  | 0.021 |  |  | 0.017 |  |  | 0.189 |
| <10% | 17 (77.3) | 24 (47.1) |  | 16 (80.0) | 25 (47.2) |  | 14 (70.0) | 27 (50.9) |  |
| ≥10% | 5 (22.7) | 27 (52.9) |  | 4 (20.0) | 28 (52.8) |  | 6 (30.0) | 26 (49.1) |  |

*P* value was calculated by chi-square test or Fisher’s exact test

TAMs, tumor-associated macrophages; SMA, smooth muscle actin
