# Supplementary material for: Prognostic Value of Tumor-Associated Macrophages According to Histologic Locations and Hormone Receptor Status in Breast Cancer
Source: PLoS One. 2015 Apr 17;10(4):e0125728. doi: 10.1371/journal.pone.0125728 (PMC4401667; doi:10.1371/journal.pone.0125728)
Supplement: S6 Table — β-catenin alteration showed a positive correlation with infiltration levels of TAMs in all compartments, and SMA expression was positively correlated with infiltration level of stromal TAMs. (DOCX) [file pone.0125728.s007.docx]

**S6 Table.** Association of TAMs with expression of epithelial-mesenchymal transition markers in triple-negative breast cancers

| **Marker** | **Intratumoral TAMs** | | ***p value*** | **Stromal TAMs** | | ***p value*** | **Total TAMs** | | ***p value*** |
| --- | --- | --- | --- | --- | --- | --- | --- | --- | --- |
|  | **Low** | **High** |  | **Low** | **High** |  | **Low** | **High** |  |
|  | **N (%)** | **N (%)** |  | **N (%)** | **N (%)** |  | **N (%)** | **N (%)** |  |
| Vimentin |  |  | 1.000 |  |  | 1.000 |  |  | 1.000 |
| <10% | 6 (42.9) | 14 (45.2) |  | 6 (46.2) | 14 (43.8) |  | 6 (42.9) | 14 (45.2) |  |
| ≥10% | 8 (57.1) | 17 (54.8) |  | 7 (53.8) | 18 (56.3) |  | 8 (57.1) | 17 (54.8) |  |
| SMA |  |  | 0.236 |  |  | 0.042 |  |  | 0.236 |
| <1% | 13 (92.9) | 23 (74.2) |  | 13 (100.0) | 23 (71.9) |  | 13 (92.9) | 23 (74.2) |  |
| ≥1% | 1 (7.1) | 8 (25.8) |  | 0 (0) | 9 (28.1) |  | 1 (7.1) | 8 (25.8) |  |
| Osteonectin |  |  | 0.236 |  |  | 1.000 |  |  | 0.236 |
| <1% | 13 (92.9) | 23 (74.2) |  | 11 (84.6) | 25 (78.1) |  | 13 (92.9) | 23 (74.2) |  |
| ≥1% | 1 (7.1) | 8 (25.8) |  | 2 (15.4) | 7 (21.9) |  | 1 (7.1) | 8 (25.8) |  |
| E-cadherin loss |  |  | 0.244 |  |  | 0.124 |  |  | 0.244 |
| <10% | 5 (35.7) | 5 (16.1) |  | 5 (38.5) | 5 (15.6) |  | 5 (35.7) | 5 (16.1) |  |
| ≥10% | 9 (64.3) | 26 (83.9) |  | 8 (61.5) | 27 (84.4) |  | 9 (64.3) | 26 (83.9) |  |
| N-cadherin |  |  | 0.717 |  |  | 0.251 |  |  | 0.277 |
| <10% | 10 (71.4) | 24 (77.4) |  | 8 (61.5) | 26 (81.3) |  | 9 (64.3) | 25 (80.6) |  |
| ≥10% | 4 (28.6) | 7 (22.6) |  | 5 (38.5) | 6 (18.8) |  | 5 (35.7) | 6 (19.4) |  |
| β-catenin alteration |  |  | 0.023 |  |  | 0.001 |  |  | 0.023 |
| <10% | 10 (71.4) | 10 (32.3) |  | 11 (84.6) | 9 (28.1) |  | 10 (71.4) | 10 (32.3) |  |
| ≥10% | 4 (28.6) | 21 (67.7) |  | 2 (15.4) | 23 (71.9) |  | 4 (28.6) | 21 (67.7) |  |

*P* values were calculated by the chi-square or Fisher’s exact test

TAMs, tumor-associated macrophages; SMA, smooth muscle actin
