# Supplementary material for: Prognostic Value of Tumor-Associated Macrophages According to Histologic Locations and Hormone Receptor Status in Breast Cancer
Source: PLoS One. 2015 Apr 17;10(4):e0125728. doi: 10.1371/journal.pone.0125728 (PMC4401667; doi:10.1371/journal.pone.0125728)
Supplement: S7 Table — β-catenin alteration was associated with high infiltration of intratumoral and total TAMs, and vimentin expression was associated with high infiltration of intratumoral TAMs. (DOCX) [file pone.0125728.s008.docx]

**S7 Table.** Association of TAMs with expression of epithelial-mesenchymal transition markers in non-triple-negative breast cancers

| **Marker** | **Intratumoral TAMs** | | ***p value*** | **Stromal TAMs** | | ***p value*** | **Total TAMs** | | ***p value*** |
| --- | --- | --- | --- | --- | --- | --- | --- | --- | --- |
|  | **Low** | **High** |  | **Low** | **High** |  | **Low** | **High** |  |
|  | **N (%)** | **N (%)** |  | **N (%)** | **N (%)** |  | **N (%)** | **N (%)** |  |
| Vimentin |  |  | 0.048 |  |  | 0.198 |  |  | 0.195 |
| <10% | 106 (98.1) | 88 (91.7) |  | 104 (97.2) | 90 (92.8) |  | 105 (97.2) | 89 (92.7) |  |
| ≥10% | 2 (1.9) | 8 (8.3) |  | 3 (2.8) | 7 (7.2) |  | 3 (2.8) | 7 (7.3) |  |
| SMA |  |  | * |  |  | * |  |  | * |
| <1% | 108 (100.0) | 96 (100.0) |  | 107 (100.0) | 97 (100.0) |  | 108 (100.0) | 96 (100.0) |  |
| ≥1% | 0 (0) | 0 (0) |  | 0 (0) | 0 (0) |  | 0 (0) | 0 (0) |  |
| Osteonectin |  |  | 0.737 |  |  | 0.739 |  |  | 0.737 |
| <1% | 104 (96.3) | 91 (94.8) |  | 103 (96.3) | 92 (94.8) |  | 104 (96.3) | 91 (94.8) |  |
| ≥1% | 4 (3.7) | 5 (5.2) |  | 4 (3.7) | 5 (5.2) |  | 4 (3.7) | 5 (5.2) |  |
| E-cadherin loss |  |  | 0.868 |  |  | 0.740 |  |  | 0.741 |
| <10% | 84 (77.8) | 73 (76.0) |  | 81 (75.7) | 76 (78.4) |  | 82 (75.9) | 75 (78.1) |  |
| ≥10% | 24 (22.2) | 23 (24.0) |  | 26 (24.3) | 21 (21.6) |  | 26 (24.1) | 21 (21.9) |  |
| N-cadherin |  |  | 1.000 |  |  | 0.575 |  |  | 0.578 |
| <10% | 101 (93.5) | 90 (93.8) |  | 99 (92.5) | 92 (94.8) |  | 100 (92.6) | 91 (94.8) |  |
| ≥10% | 7 (6.5) | 6 (6.2) |  | 8 (7.5) | 5 (5.2) |  | 8 (7.4) | 5 (5.2) |  |
| β-catenin alteration |  |  | 0.010 |  |  | 0.057 |  |  | 0.035 |
| <10% | 103 (95.4) | 81 (84.4) |  | 101 (94.4) | 83 (85.6) |  | 102 (94.4) | 82 (85.4) |  |
| ≥10% | 5 (4.6) | 15 (15.6) |  | 6 (5.6) | 14 (14.4) |  | 6 (5.6) | 14 (14.6) |  |

*P* values were calculated by the chi-square or Fisher’s exact test

TAMs, tumor-associated macrophages; SMA, smooth muscle actin

* could not be calculated
